# Hierarchical Decomposition for Betweenness Centrality Measure of Complex Networks

**DOI:** 10.1038/srep46491

**Published:** 2017-04-20

**Authors:** Yong Li, Wenguo Li, Yi Tan, Fang Liu, Yijia Cao, Kwang Y. Lee

**Affiliations:** 1 College of Electrical and Information Engineering, Hunan University, Changsha 410082, China; 2 School of Information Science and Engineering, Central South University, Changsha 410083, China; 3 Department of Electrical and Computer Engineering, Baylor University, Waco 76798-7356, Texas, USA

## Abstract

Betweenness centrality is an indicator of a node’s centrality in a network. It is equal to the number of shortest paths from all vertices to all others that pass through that node. Most of real-world large networks display a hierarchical community structure, and their betweenness computation possesses rather high complexity. Here we propose a new hierarchical decomposition approach to speed up the betweenness computation of complex networks. The advantage of this new method is its effective utilization of the local structural information from the hierarchical community. The presented method can significantly speed up the betweenness calculation. This improvement is much more evident in those networks with numerous homogeneous communities. Furthermore, the proposed method features a parallel structure, which is very suitable for parallel computation. Moreover, only a small amount of additional computation is required by our method, when small changes in the network structure are restricted to some local communities. The effectiveness of the proposed method is validated via the examples of two real-world power grids and one artificial network, which demonstrates that the performance of the proposed method is superior to that of the traditional method.

Betweenness centrality (BC) is a fundamental and useful index for measuring the importance of a vertex within a graph, because it is primarily defined as the ratio of shortest paths between vertex pairs that pass through the vertex of interest^1^,^2^. BC has been applied in many complex networks. For instances, it can be used to detect the community structure of biological networks^3^, to analyze the topological structure of social networks^4^ and protein networks^5^, to control the synchronization of air networks^6^,^7^, and to enhance power grid robustness against malicious attacks^8^,^9^.

Measuring BC requires to calculate the shortest paths between all pairs of vertices in a graph, and the early method, i.e., the Floyd method^10^, requires *O(n*^3^) time, where *n* is the number of vertices. This computation becomes prohibitively expensive, especially for the dynamic online analysis such as live traffic estimation and navigation^11^ and epidemic control in large-scale communication networks. Since BC was introduced by Anthonisse^12^ and Freeman^13^, many related methods have been developed to increase the speed of BC computation. Brandes proposed a fast method that uses vertex pair-dependency to compute the BC of large networks^14^. Similarly, another fast method was developed by Newman^4^ to analyze scientific collaboration networks, which requires *O(nm*) time, where *m* is the number of edges. Puzis *et al*. proposed two complementary heuristics to enhance the BC computation speed^15^. Pontecorvi and Ramachandran^16^ introduced a fast method for fully dynamic BC computation. Several approximate BC methods have also been presented to improve calculation speed by using randomized methods^17^,^18^,^19^. The accuracy of these approximate methods may decrease as the size of the network increases. Moreover, modified Brandes’ methods have also been presented to calculate the variants of BC, such as flow betweenness^20^ and random-walk betweenness^21^. In all of the aforementioned methods, the global structural information is required to compute the shortest paths between all pairs of vertices in a graph, hence, the computations become prohibitive for large-scale networks. For example, Brandes’ and Newman’s methods both require *O(nm*) time. Therefore, most of the current methods are unsuitable for online application.

Many real-world systems, such as social networks^1^,^4^, electric power grids^9^, communication networks^6^, and biological networks^3^,^5^, have a community structure, which consists of subsets of vertices that are densely connected to each other but sparsely connected to the rest of the network. In such a structure, the strong ties (intracommunity edges) are primarily along the shortest paths between the vertices of the same communities; conversely, the weak ties (intercommunity edges) that connect two communities are covered by many shortest paths between the nodes of different communities. Many methods^22^,^23^,^24^,^25^,^26^,^27^,^28^,^29^,^30^,^31^, inspired by this pattern, have been proposed to detect the community structure of networks^22^. Particularly, Girvan *et al*. proposed one elegant method^3^ for community detection that iteratively deletes the highest betweenness edges and hierarchically decomposes the networks. Conversely, the BC calculation could be simplified by using the local structural information of these communities in a graph.

In this article, a new decomposition method that uses the local structural information from different communities is proposed to speed up the betweenness calculation for complex networks with a community structure. This method is proved to be rigorously valid, and can be applied to any network with community structure, inspective of any community detection methods. This improvement is much more evident for those networks that has numerous communities, uniform community size and strong hierarchy structure. Our analysis shows that the runtime complexity for such weighted and unweighted networks with known community structure can be even reduced from 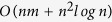 and *O(nm*) to 
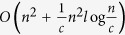
 and *O(nm*) to *O(n*^2^), respectively, where *c* is the number of communities of the networks. Furthermore, the proposed method features a parallel structure, hence, its computation speed can be enhanced via parallel calculation. Moreover, when small changes in the network structure are restricted to some local communities, e.g., an line outage in power grids, only some additional computations are required for our method while a complete recalculation is needed by other methods. Therefore the proposed method is suitable for real-world, online BC-related application. The proposed method is compared with the traditional methods, and the results validate its superiority.

## Methods

### Hierarchical decomposition modelling

**Figure 1:**
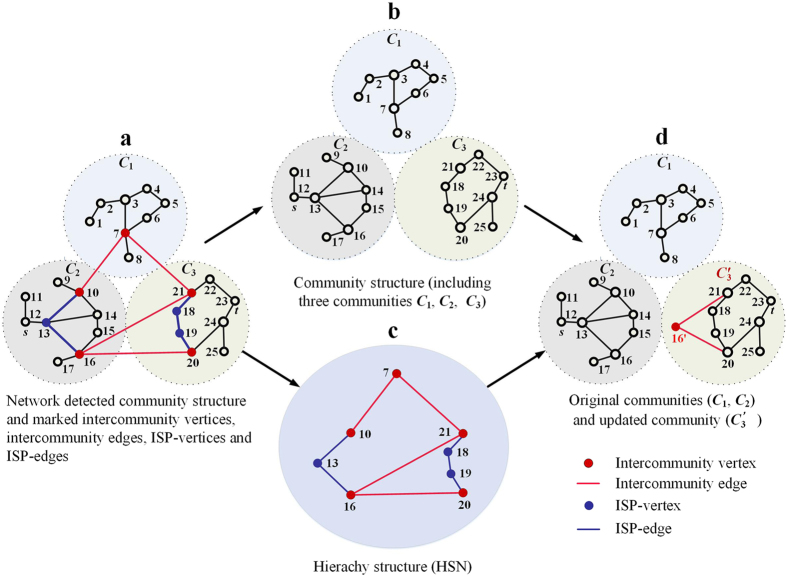
Hierarchical community structure and updated communities. (**a**) A network detected community structure and marked intercommunity vertices, intercommunity edges, ISP-vertices and ISP-edges. (**b**) The community structure including three communities (*C*_1_, *C*_2_, *C*_3_). (**c**) The hierarchy structure (HSN). (**d**) The original communities (*C*_1_, *C*_2_) and the updated community (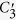). For the community *C*_3_, the condition that the shortest paths (*e*_21,18_ + *e*_18,19_ + *e*_19,20_) between the two intercommunity vertices (*v*_20_, *v*_21_) in *C*_3_ are equal or greater than the shortest paths (*e*_21,16_ + *e*_16,20_) in the HSN, is satisfied, thus the community *C*_3_ is updated to 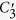 by copying those vertices and edges (*v*_20,_ *v*_21,_ *e*_21,16_ and *e*_16,20_) through which the shortest paths between the two intercommunity vertices (*v*_20_, *v*_21_) pass in the HSN.

Here we illustrate the model of hierarchical community structure and the updated community with a simple network composed of three communities, as shown in Fig. 1. Community of network consists of subset of vertices that are densely connected to each other but sparsely connected to the rest of the network such as *C*_1_, *C*_2_ and *C*_3_ in Fig. 1. The intercommunity vertices (*v*_7_, *v*_10_, *v*_16_, *v*_20_, *v*_21_) are the terminal vertices of the intercommunity edges (*e*_7,10_, *e*_7,21_, *e*_16,21_, *e*_16,20_). Those vertices (*v*_13_, *v*_18_, *v*_19_) and the edges (*e*_10,13_, *e*_13,16_, *e*_20,21_) that lie on the shortest paths between the intercommunity vertices of the same community (ISP) are called the ISP-vertices and the ISP-edges, respectively. Community structure consists of independent communities, as shown in Fig. 1(b). The hierarchy structure is the top level of a network and is also called the hierarchical subnet (HSN), which consists of intercommunity edges, intercommunity vertices, ISP-vertices and ISP-edges, as shown in Fig. 1(c).

The proposed decomposition approach for BC calculation is to turn the BC computation in global network into the computation in hierarchical subnet (HSN) and every independent community (more detailed in Algorithm). However, the BC computation within some original communities will generate calculation errors if the communities satisfy the condition that the shortest path lengths between the intercommunity vertex pairs of the community are equal or greater than that of the HSN (see Supplementary Lemma S1). To solve this problem, the original communities satisfying the condition must be updated.

Those communities satisfying the aforementioned condition are updated by copying those vertices and edges through which the shortest paths between the intercommunity vertex pairs pass in the HSN into the community, such as the updated community 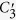 from *C*_3_ shown in Fig. 1(d). For the communities that do not satisfy the condition, the update is not needed, e.g., the community *C*_1_ and *C*_2_ shown in Fig. 1(d).

**Algorithm:** For a network with a hierarchical community structure, the HSN plays a vital role because it bridges all communities, especially the intercommunity vertices and the intercommunity edge of the HSN. This indicates that the betweenness calculation for a network with a hierarchical community structure can be decomposed into two main stages by the integrated use of local and global information. The first stage involves searching for the shortest paths and calculating BC within local communities and the HSN independently. In the second stage, BC is calculated for every pair of vertices from different communities via the HSN. In the proposed method, we use breadth-first search to search for the shortest paths of unweighted networks and Dijkstra’s algorithm for weighted networks, and Brandes’ method is used to calculate BC in every community and the HSN. More specifically, the proposed method for computing betweenness is stated as follows (also see the pseudo-code of the method in Table 1):

**Step 1.** Mark the intercommunity edges and the intercommunity vertices based on the community structure. Note that the structures of some networks are unknown in advance. In this case, the community structure of the network is detected by using a well-known fast method^22^,^23^,^24^,^25^,^26^,^27^,^28^,^29^.

**Step 2.** Isolate each community from the other communities. Then, search for and store the shortest paths for all intercommunity vertex pairs in each community. Mark ISP-vertices and ISP-edges.

**Step 3.** Distill the hierarchy structure to construct HSN by using the vertices and edges marked in Steps 1 and 2. Search and store the shortest paths for all intercommunity vertex pairs in HSN, calculate the BC with Brandes’ method and store the number of the shortest paths between each intercommunity vertex pair for those vertices and edges through which the shortest paths pass.

**Step 4.** For any two intercommunity vertices in each community, if the shortest path length between them in the community (in Step 2) is equal or greater than that in the HSN (in Step 3), then update the community by copying those vertices and edges through which the shortest paths between them pass in the HSN into the community, as illustrated in Fig. 1(d).

**Step 5.** Search and store the shortest paths for all vertex pairs in each community except the intercommunity vertex pairs, calculate the BC with Brandes’ method and store the number of the shortest paths between each intercommunity vertex pair for those vertices and edges through which the shortest paths pass.

**Step 6.** Based on the data saved in Steps 3 and 5, calculate the shortest paths for any vertex pairs of different communities except the intercommunity vertex pairs via the HSN and update the BC for those vertices and edges through which the shortest paths pass.

The Step 6 of the proposed algorithm can be further decomposed (Supplementary Fig. S1), and the BCs of vertices and edges lying in the shortest paths between the vertices of different communities, can be updated according to the Supplementary equations S6, S7 and S8.

The computational efficiency of the proposed decomposition method is superior to the computational efficiency of other methods^4^,^10^,^14^,^16^ that are based on the global structural information, because only the local structural information from the communities and the HSN of network are required. Furthermore, from the mathematical analysis and proof (see Supplementary Method Section S1.2, S1.3), it is shown that the proposed method described by Steps 1–6 is rigorous and valid, and can be applied to any networks with hierarchical community structure, inspective of any community detection methods.

### Computational complexity

The preprocessing runtime in Step 2 is *w*_i_*m*_i_ and 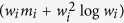 for unweighted and weighted networks, respectively, where *m*_*i*_ and *w*_*i*_ are the number of edges and intercommunity vertices of the community *C*_*i*_, respectively. The runtime of Step 3 for unweighted and weighted networks is *wq* and (*wq* + *w*^2^log *w*), respectively, where *w* and *q* are the numbers of intercommunity vertices and edges in the HSN, respectively. For a community *C*_*i*_, the runtime of the BC computation inside *C*_*i*_ is (*n*_i_ − *w*_i_)*m*_i_ and [(*n*_i_ − *w*_i_)*m*_i_ + (*n*_*i*_ − *w*_*i*_)^2^log (*n*_*i*_ − *w*_*i*_)] for the unweighted network and the weighted network, respectively, where *n*_*i*_ is the number of vertices of the community *C*_*i*_ (in Step 5).

The runtime of the BC computation from a non-intercommunity vertex of any a community *C*_*i*_ to all vertices of other communities is (*n* − *n*_*i*_) for both the unweighted and the weighted networks (in Step 6), where *n* is the number of vertices in the network. Then, for all vertices of *C*_*i*_, the runtime is (*n*_*i*_ − *w*_*i*_)(*n* − *n*_*i*_). Furthermore, the BC computation runtime for all communities of an unweighted network can be calculated by


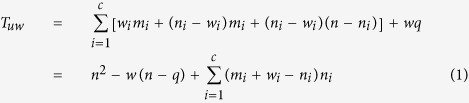


Where 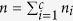, 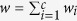, and *c* is the number of communities of the network.

Similarly, for a weighted network, the BC computation runtime for all communities is given by


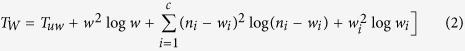


Then, we analyze two particular networks: the one without hierarchical community structure, and the other one with hierarchical community structure of many communities which possess the same number of vertices, edges, intercommunity vertices, ISP-vertices and ISP-edges. For the former one, the whole network is a community, and thus the number of community is equal to 1 and w = 0, *T*_*uw*_ and *T*_*w*_ reach the maximum value at (*T*_*uw*_)_max_ = *mn* and (*T*_*w*_)_max_ = *mn* + *n*^2^log *n*, respectively. For the latter one, 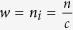, 
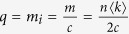
, *T*_*uw*_ and *T*_*w*_ can be simplified as follows:


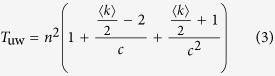



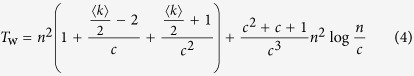


Where 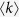 is average degree of the network.

If 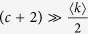, 
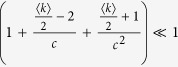
, thus, *T*_uw_ and *T*_w_ can be approximated as follows:


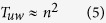



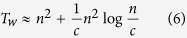


The above analysis shows that the computation complexities of the proposed BC method are 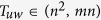 and 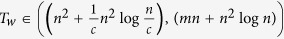 for the unweighted and weighted networks with known community structure, respectively. More specifically, the computation complexity is related to the hierarchical community characteristics of network: more numerous communities, more uniform community size and stronger hierarchical structure (smaller HSN) result in less computational time. For the networks with unknown community structure, community detection time must be considered in our method.

### Extension to a dynamic environment and the parallel computation

In the dynamic environment, there could be frequent small changes of network topological structure which are restricted to some local communities. For instance, a line outage is likely to be trigged due to a random failure in power grids. In such a dynamic environment, the BC of networks needs frequent online update, for example, reevaluating the capacities of power transmission lines or traffic capacities in transportations.

**Figure 2:**
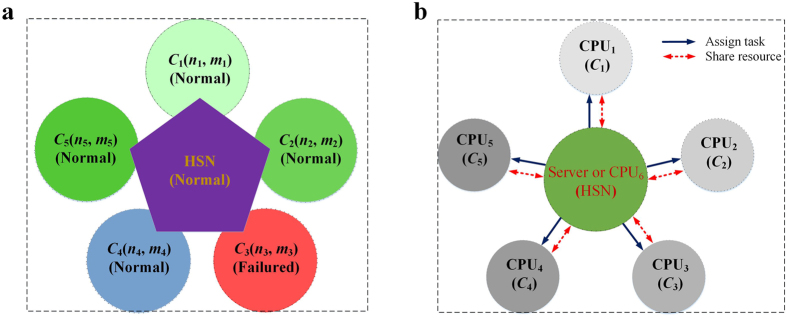
The dynamic computation and parallel computation. In (**a**) the dynamic computation, the red community *C*_3_ represents the failure area due to random failures or perturbs. In (**b**) the parallel computation, the HSN is the centrality of task assigning and information sharing, and the computational tasks of server and CPUs are independent with each other.

In this aspect, a complete recalculation is required by other methods and this is time-consuming. In contrast, much less time is needed for our method in such a dynamic environment. For example, as shown in Fig. 2(a), only the BC within *C*_3_ and between *C*_3_ and *C*_*i*_ (*i* = 1, 2, …, 5) are recalculated, and the total runtime complexity is 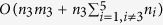 which is approximately equal to 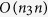, where 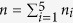, 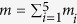. Hence, from equation (5), the runtime of the dynamic betweenness calculation is approximately decreased to *n*_3_*/n* times the runtime of the static betweenness calculation.

Our method also has a natural advantage in parallel calculation. The computational resources, including the memories and procedures required by our method, can be conveniently divided in a parallel manner, because the information used to search for the shortest paths comes from the independent communities and HSN. Furthermore, the data sets stored in Steps 3 and 5, which are used to calculate the betweenness, are also independent. A simple parallel implementation of our method is the allocation of computing resources according to the communities and the HSN. For instance, as shown in Fig. 2(b), a server and five CUPs are assigned to calculate the BC for a given network with five communities, and their computational tasks are assigned as follow:

1. The server detects and isolates the communities of network, marks the intercommunity edges and vertices, assigns computational tasks according the size and number of communities and send the community information to other CPUs;
2. Each CPU searches the shortest paths for all intercommunity vertex pairs in its community, marks and shares ISP-vertices and ISP-edges with the server.
3. The server constructs HSN, searches the shortest paths for HSN and shares the information of shortest paths with each CPU; each CPU calculates the BC and shares the shortest paths of its community with the server.
4. CPU_*i*_ computes the BC and shortest paths within community *C*_*i*_ and between *C*_*i*_ and (*C*_*i+*1_ and *C*_*i+*2_) (if *i* > 5, *i* = *i*−5) and shares those computational information with the server, the server updates BC for all vertices and edges.

For the above analysis, one can see that the main task of CPU_*i*_ is to compute the BC of vertices and edges in its community *C*_*i*_ and between *C*_*i*_ and (*C*_*i+*1_ and *C*_*i+*2_), while the server coordinates tasks primarily, calculates BC of the HSN and updates BC for all vertices and edges. The computational tasks should be uniformly assigned in practical application for avoiding cask effect.

## Results

### Hierarchical community structure of test networks

We tested the proposed method on one artificial network with a known community structure and two representative real-world networks with unknown community structures, i.e., the Henan provincial power grid and the Gansu provincial power grid in China^32^,^33^. The artificial network consists of 9 random subnets (communities) in which each vertex has 16 edges on average. Each subnet has the same number of vertices and 3 interconnected intercommunity vertices. The Henan power grid consists of 310 vertices (nodes) and 932 edges, and the Gansu power grid has 1569 vertices and 4326 edges. The vertices represent transformer substations or power plants, and the edges denote their interconnections^34^,^35^.

The community structure of the artificial network is known in advance, but the community structure of each power grid needs to be detected before applying our method. Three detection methods are presented to detect the community structure of power grids. The first one is the voltage information based detection method (VIDM). The community structure information of a power grid can be detected via the voltage grade of the vertices and edges, because a power grid is designed and operated according to the voltage grade. Therefore, the information-theory based method^31^ is used to divide each power grid into different communities by deleting the edges with high-level voltage (500 kV for the Henan power grid, 750 kV and 330 kV for the Gansu power grid). By using this method, the Henan and the Gansu power grids are divided into 9 and 5 communities, respectively. The second one is the geographical information based detection method (GIDM), which is used to divide the Henan and the Gansu power grids into 15 and 13 communities, respectively. The third one is the detection method^24^ proposed by Radicchi *et al*. (RCDM). It is an effective and efficient approach to determine the community structures of networks that yields the correct number of communities without prior knowledge. By using the RCDM, the Henan and the Gansu power grids are divided into 9 and 6 communities, respectively, as shown in Table 2.

### Computation accuracy

**Figure 3:**
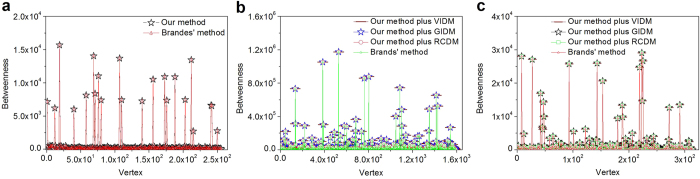
Calculation validity. (**a**) The betweenness of each vertex in the artificial network. (**b**) The betweenness of each vertex in the Gansu power grid. (**c**) The betweenness of each vertex in the Henan power grid. The vertical axes means the betweenness value of each vertices, and the horizontal axis expresses unique number given to each node. The number represents the ID of each vertex.

We test the effectiveness of our method on one artificial network with a known community structure and two real-world power grids with unknown community structures. The performance of our method is compared with the performance of the well-known Brandes’ method. As shown in Fig. 3 (and see Supplementary Fig. S3), the BCs of each vertex and edge obtained by Brandes’ method and our method perfectly match each other. Taking the results of Brandes’ method as a reference, we also calculate the relative errors of our method. The accumulated relative betweenness errors of vertices and edges are both close to zero. The results indicate that the proposed method is rigorous and valid, and our method is accurate regardless of network partition methods (see also Method and Supplementary Method Section S1.2 and S1.3 for a detailed analysis and proof).

### Computation efficiency

**Figure 4:**
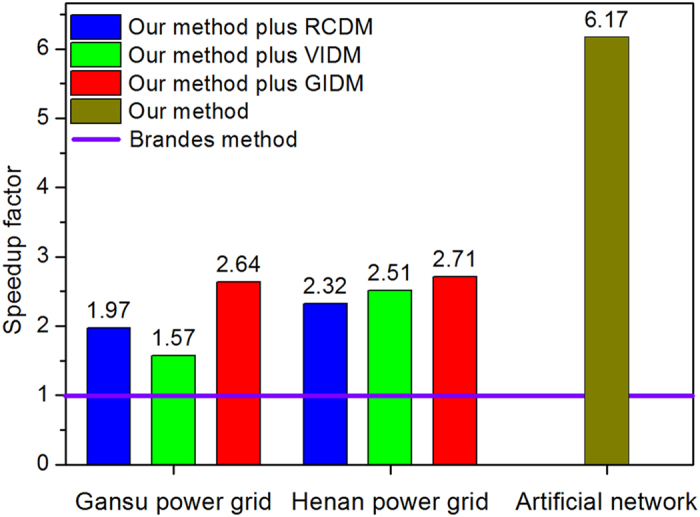
Speedup factor comparison for networks. For the artificial network with a known community structure, we compare the computational speed (speedup factor) of our method with Brandes’ method; and for the Henan and the Gansu power grids with unknown community structure, we compare the speedup factors of our method plus three detection methods (VIDM, GIDM and RCDM) combined with Brandes’ method.

**Figure 5:**
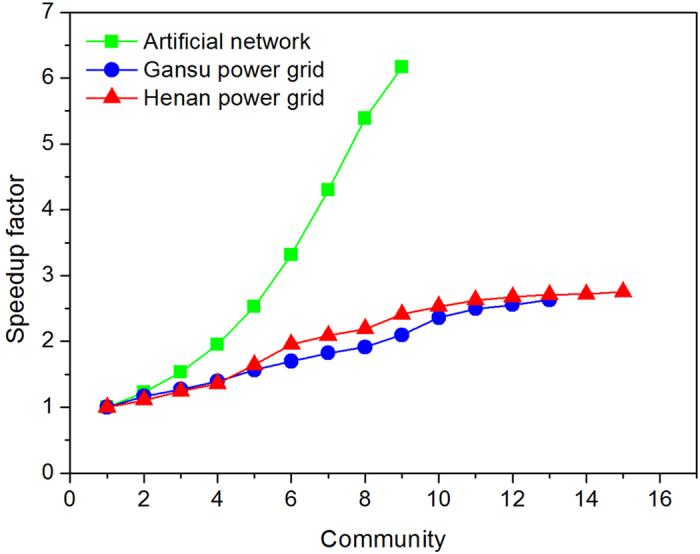
Effect of the number of communities on computational efficiency. For the artificial network, the Henan and the Gansu power grids, their speedup factors rise with the increase of the number of their partitioned communities, meanwhile their sizes and structures are kept unchanged in experiments. For Henan and Gansu power grids with unknown community structure, we detect their community structures by using GIDM. We obtain different number of communities by treating the whole of some communities as a new larger community.

**Figure 6:**
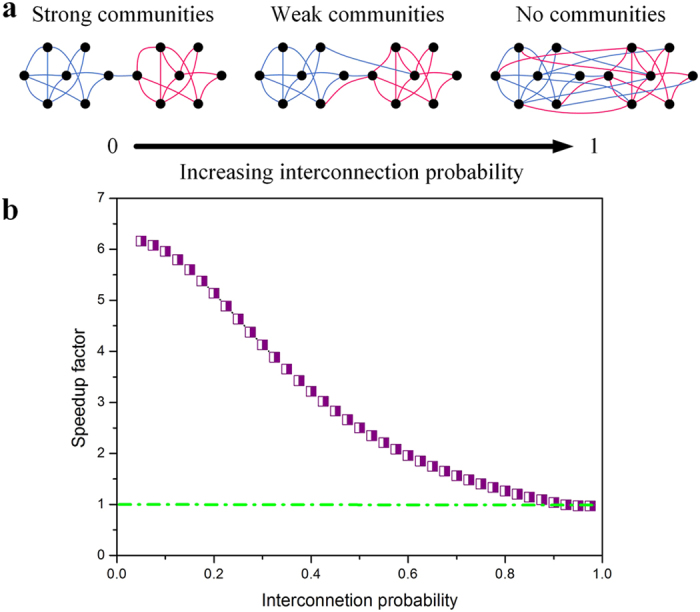
Effect of the community structure strength on the computational efficiency. The size and community structures of the artificial network are kept unchanged in experiments. (**a**) With reference to ref. 36, we illustrate the findings with a simple network composed of two communities, where the community structure is modulated by the interconnection (intercommunity) edges starting from two separated random graphs. (**b**) The speedup factor decreases by degrees when the artificial network changes from the strong community structure to no community structure.

**Figure 7:**
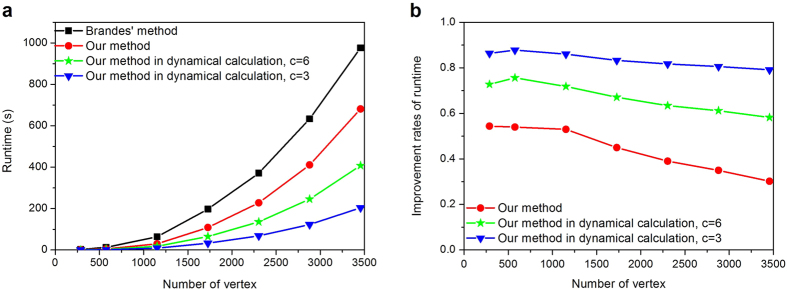
Effect of the network size on the computational efficiency in the artificial networks. (**a**) Runtimes for computing the betweenness of the artificial network with 288 to 3456 vertices. (**b**) Improvement rates of runtime. In all cases, the number of communities is unchanged. Parameter *c* represents the number of communities whose topological structures are changed. We select a community and remove three edges randomly for simulating local failures each time, until the number of failure communities, *c*, satisfies the experimental requirements.

The computational performance of our method is further tested on the artificial network and the two power grids. The corresponding results are shown in Figs 4, 5, 6, 7.

In Figure 4, for the artificial network, our method can speed BC calculation up to 6.17 times, as compared with Brandes’ method. For the Gansu power grid which is partitioned into 5, 6 and 13 communities by VIDM, GIDM, and RCDM, respectively, as shown in Table 2, and the speedup factors of the hybrid methods, defined as the ratio of runtime of Brandes’ method to that of the hybrid methods, are 1.57, 1.97 and 2.64, respectively. Even if the number of communities is the same, for instance, the Henan power grid is divided into 9 communities by VIDM and RCDM, respectively, as shown in Table 2, the asymmetrical community sizes bring about diverse computation speeds (the speedup factors are 2.32 and 2.51, respectively) (see also equation (1) and equation (2)).

The effect of the number of partitioned communities on the computational efficiency is compared, as shown in Fig. 5. This figure shows that the increasing numbers of communities lead to larger computational speedup factors (see also equation (1) and equation (2)), since the speedup factor curves of the artificial network, the Henan and Gansu power grids rise.

The strong community structure^36^, as illustrated in Fig. 6(a), is another factor that affects the computational efficiency of the proposed method. The strong (or weak) community structure means sparser (or denser) intercommunity edges between communities, namely, smaller (or greater) interconnection probability between communities. As shown in Fig. 6(b), one can see that the speedup factor gradually drops to 1, when the network structure is changed from strong community structure to no community structure (increasing interconnection probability). This is because a weaker community structure brings about a greater size of the hierarchical subnet (HSN) and results in a greater computational complexity (see also equation (1) and equation (2)).

### Runtime improvement rates on network size and dynamic environment

In Figure 7, the artificial network consists of 9 random subnets (communities). Each subnet has 32 vertices randomly interconnected including three interconnected intercommunity vertices. Each vertex possesses 6 edges on average. Then we increase the number of vertices from 288 to 3456 with a step of 576 while keeping the number of communities fixed. Note that some edges are added to ensure the average degree is fixed during the modification. As shown in Fig. 7(a), the runtime of both our and Brandes’ method grow with increasing number of vertices in power function as theoretical predictions (i.e., *O(n*^2^) and *O(nm*), respectively), furthermore, the runtime of our method is significantly lower than that of Brandes’ method, which indicates the faster computational speed of our method. The runtime of our method in the dynamic environment is also shown in Fig. 7(b). The improvement rate of runtime is defined as a ratio of the runtime difference between Brands’ and our methods to the runtime of Brands’ method. Figure 7(b) shows that the computation time in the dynamic environment is significantly lower than the runtime required for Brandes’ method. This is because that the previous BC information can be used to update the BC, when the inner structures of the communities are changed. Figure 7 also shows that the fewer the number of communities is changed, the faster the calculation speed is. This means that if more communities are changed, more calculation is required to update the BC. Figure 7(b) shows the improvement rates of the runtime, which indicates that the improvement rates in the dynamic environment is higher than that of Brandes’ method. The fewer the number of communities is changed, the higher the improvement rate is. Moreover, Fig. 7 shows that the improvement rate is decreased with the increase of the network size, which occurred because more computation time is required to search for the shortest paths of each vertex-pair in each community, when the number of communities is kept the same and the size of each community becomes increasingly larger. Notably, the improvement rate decreases slowly in a dynamic environment. This good performance is gained since that only additional computation is needed with the change of the community structure.

### Investigation of our methods on networks of different density

As a first, we give a theoretical analysis on the effects of the density of both unweighted and weighted networks on the speedup factor of our method. From Brands’ method and the equations (3, 4), the theoretical speedup factors, for unweighted and weighted networks with homogenous community structure, can be derived as follows:


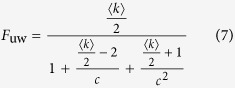



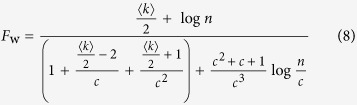


If 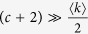, then, *F*_uw_ and *F*_w_ can be approximated as follows:


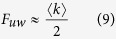



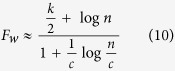


**Figure 8:**
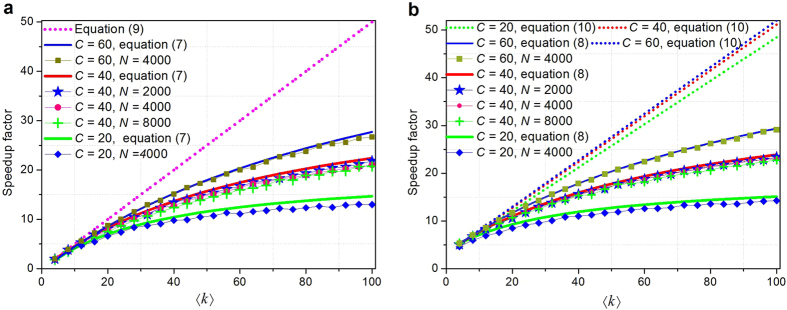
Speedup factor vs average degree. (**a**) The unweighted networks (**b**) The weighted networks. In each tested artificial network, each subnet has the same number of vertices and three interconnected intercommunity vertices and edges. The average degree of each artificial network is varied by increasing randomly the same number of edges in each subnet. Parameters *C, N* represent the number of total communities and vertices of these artificial networks respectively. The networks are weighted with reference to ref. 37.

Secondly, we validate the above analysis on a larger set of artificial networks by varying average degrees for unweighted and weighted networks. As shown in Fig. 8, the speedup factors grow linearly with average degree as theoretical predictions of equation (9) and (10), when the networks are very sparse (in the initial stages). For both unweighted and weighted networks with the same number of communities, the speedup factors become increasingly hyperbolic with the growth of average degrees, which agree well with the theoretical results of equation (7) and (8). From the comparison of Fig. 8(a) and Fig. 8(b) (or equation (7) and (8)), one can see that the weighted networks have better speedup performance than unweighted networks.

### Parallel implementation of our methods

**Figure 9:**
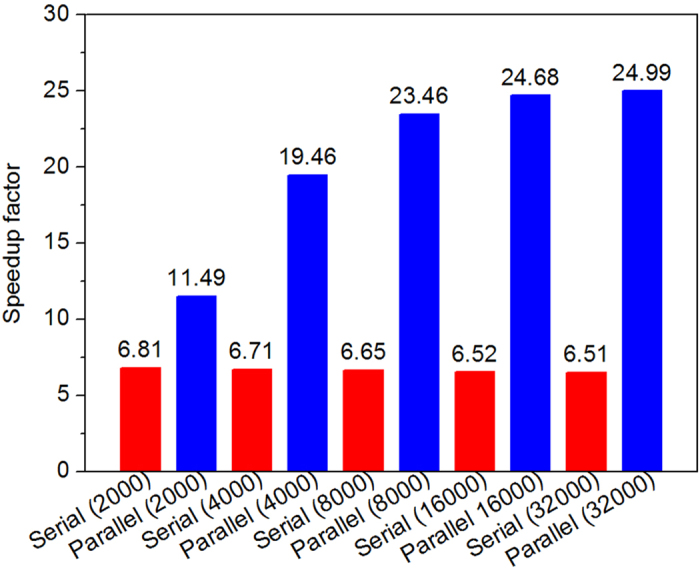
The speedup factor of parallel computation. In the five tested artificial networks, each network consists of 20 communities (subnets), each subnet has the same number of vertices and three interconnected intercommunity vertices and edges, and the average degrees of each networks is 20. The numbers in the brackets represent size of the networks.

Here, a representative example of the parallel implementation of our method is provided. The performance of parallel computation is tested on five artificial networks by six computers as shown in Fig. 9. The tasks of the six computers (CPUs or server) are divided as shown in Table 3, and the six computers compose of a local area network through a switch and use TCP/IP as their communication protocol. The tested results indicate that our method can further speed up the BC calculations by parallel computation. With the growth of the network size, the speedup performances are more prominent, because the essential communication and previous pretreatment cost is much less as compared with the total computational time.

## Discussion

In this article, we propose a new decomposition method to enhance the efficiency of the betweenness calculation for networks with community structures. This method (including steps 1–6 in Methods) is rigorous and valid, and can be applied to any networks with community structure, inspective of any community detection methods (more detailed mathematical analysis and proof in Supplementary Method Section S1.2 and S1.3).

The computational efficiency of our method is related to the hierarchical community characteristics of networks. Namely, more uniform community size, more numerous communities and stronger hierarchical structure (smaller HSN) in networks will result in less computational complexity of our method (see equation (1), equation (2) and Figs 4, 5, 6). For such networks, if 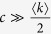, the runtime of our method for the weighted networks can be even reduced from 
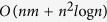
 to 
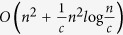
, and the runtime for the unweighted networks can be reduced from *O(nm*) to *O(n*^2^), where *n, m* and *c* are the numbers of vertices, edges and communities, respectively (detailed in the computational complexity of Method Section). Our method can also speed up the betweenness calculation for other atypical network with community structure. Thus, our method shows better performance than traditional methods. Moreover, the time complexity in a dynamic environment can also be effectively reduced by using our method.

For networks with an unknown community structure, it is necessary to detect the community structure of the networks, and the runtime of our method must include the runtime of detecting the community structure. Indeed, the community partition quality of detection methods influences directly the computational performance of our method (see complexity analysis in Method Section), and unsuitable detection methods may worse the computational speed of our method. However, if the community structure is known in advance, our method is much faster than other methods. In all case studies, our method plus other detection methods (including GIDM, VIDM, RCDM) is still much faster than traditional methods. For a larger scale network with unknown community structure, our hybrid method may be inapplicable because of excessive division cost from the detection methods such as RCDM. Furthermore, our method is naturally suitable for parallel calculation because the shortest path and betweenness within each community can be independently calculated. It is promising that our method can also help to enhance the calculation speed of the variants of betweenness and centrality, such as stress centrality^1^,^14^.

## Additional Information

**How to cite this article:** Li, Y. *et al*. Hierarchical Decomposition for Betweenness Centrality Measure of Complex Networks. *Sci. Rep.* **7**, 46491; doi: 10.1038/srep46491 (2017).

**Publisher's note:** Springer Nature remains neutral with regard to jurisdictional claims in published maps and institutional affiliations.

## Supplementary Material

Supplementary Information

## Figures and Tables

**Table 1:** The pseudo-code of the proposed method.

**Algorithm 1:**
**Input:** G(*V, E*): A network with community structure;
**Output:** *VB*[*v*], *EB*[*e*]: BC of vertex and edge;
*C*_0_ ← 0: The number of communities;
*VB*[*v*] ← 0, *v* ϵ *V*; *EB*[*e*] ← 0, e ϵ *E*;
**if** The community structure of the network is unknown **then**
Detect the community structure;
**end if**
*C*_0_ ← The number of communities;
Mark the intercommunity edges and vertices;
**for** i = 1 **to** *C*_0_ **do**
search and store the shortest paths for all intercommunity vertex pairs in *C*_*i*_;
**end for**
Mark all ISP-vertices and ISP-edges; by using Brands’ method;
Construct HSN and search and store the shortest paths in HSN;
**for** i = 1 **to** *C*_0_ **do**
Update the community *C*_*i*_;
**end for**
**for** i = 1 **to** *C*_0_ **do**
**for** j = 1 **to** *n*_*i*_ **do**//*n*_*i*_ is the number of vertices of community *C*_*i*_
**for** *k* = 1 **to** *n*_*i*_ **do**
Search the shortest paths for all vertex pairs (*i, j*) and calculate the BC in *C*_*i*_ by using Brands’ method;
**end for**
**end for**
**end for**
**for** i = 1 **to** *N* **do**
**for** j = 1 **to** *N* **do**
**if** *C*_*i*_ ≠ *C*_*j*_ then
Calculate the shortest paths for all vertex pairs (*i, j*);
Update the BC for the vertices and edges lying in the shortest paths;
**end if**
**end for**
**end for**

**Table 2:** Community structures of the power grids.

Power grids	Detection algorithm	Total	HSN	*C*_1_	*C*_2_	*C*_3_	*C*_4_	*C*_5_	*C*_6_	*C*_7_	*C*_8_	*C*_9_	*C*_10_	*C*_11_	*C*_12_	*C*_13_	*C*_14_	*C*_15_
Henan	GIDM (N/E)	310	49	26	12	30	10	8	15	15	20	20	20	43	44	23	13	11
		932	148	77	32	86	29	21	38	41	46	48	52	129	131	62	35	30
	VIDM (N/E)	310	24	26	27	40	23	40	63	44	23	24	\	\	\	\	\	\
		932	62	77	79	122	67	104	191	131	62	70	\	\	\	\	\	\
	RCDM (N/E)	310	28	26	27	20	20	23	40	107	23	24	\	\	\	\	\	\
		932	74	77	79	54	60	67	104	326	62	70	\	\	\	\	\	\
Gansu	GIDM (N/E)	1569	109	185	118	42	100	185	96	45	58	90	121	89	68	92	\	\
		4326	562	423	255	97	242	520	250	109	126	209	262	223	166	215	\	\
	VIDM (N/E)	1569	12	185	136	104	103	1041	\	\	\	\	\	\	\	\	\	\
		4326	18	445	339	260	276	2993	\	\	\	\	\	\	\	\	\	\
	RCDM (N/E)	1569	12	185	136	104	103	671	370	\	\	\	\	\	\	\	\	\
		4326	18	445	339	260	276	2101	891	\	\	\	\	\	\	\	\	\

For each power grid, we detect its community structures by using the three detection algorithms (GIDM, VIDM and RCDM) and show the network size (Total) that includes the numbers of nodes (*N*) and edges (*E*). The HSN (intermediary subnet) is a subnet consisting of intercommunity edges, intercommunity vertices, ISP-vertices and ISP-edges; the parameter *C*_*i*_ represents the *i*^th^ partitioned community.

**Table 3:** The task partition of parallel computation.

Task	CPU_1_	CPU _2_	CPU _3_	CPU _4_	CPU _5_	Server
Internal	C_1_, C_2_, C_3_, C_4_	C_5_, C_6_, C_7_, C_8_	C_9_, C_10_, C_11_, C_12_	C_13_, C_14_, C_15_, C_16_	C_17_, C_18_, C_19_, C_20_	HSN
External	C_i_ ↔ C_j_ (i = 1, 2, 3, 4) (j = 5, 6, …, 12)	C_i_ ↔ C_j_ (i = 5, 6, 7, 8) (j = 9, 10, …, 16)	C_i_ ↔ C_j_ (i = 9, 10, 11, 12) (j = 13, 14, …, 20)	C_i_ ↔ C_j_ (i = 13, 14, 15, 16) (j = 17, 18, …, 4)	C_i_ ↔ C_j_ (i = 17, 18, 19, 20) (j = 1, 2, …, 8)	update BC

For a computer (CPU), its internal task is to calculate the BC in and between its five communities assigned by the server, while its external task is to compute the BC between its communities and the communities of other CPUs (*C*_*i*_ ↔ *C*_*j*_). The main task of the server is to calculate the BC in the HSN, assign the tasks to other computers and update the BC.
