# Supplementary material for: Hierarchical Decomposition for Betweenness Centrality Measure of Complex Networks
Source: Sci Rep. 2017 Apr 20;7:46491. doi: 10.1038/srep46491 (PMC5397849; doi:10.1038/srep46491)
Supplement: Supplementary Information [file srep46491-s1.doc]

**Hierarchical Decomposition for Betweenness Centrality Measure of Complex Networks**

**(Supplementary information)**

Yong Li^1,*^, Wenguo Li ^1^, Yi Tan^1,*^, Fang Liu^2^, Yijia Cao^1^, and Kwang Y. Lee^3^

^1^College of Electrical and Information Engineering, Hunan University, Changsha 410082, China.

^2^School of Information Science and Engineering, Central South University, Changsha 410083, China.

^3^Department of Electrical and Computer Engineering, Baylor University, Waco, Texas 76798-7356, USA

Correspondence authors: yongli@hnu.edu.cn (Y.L.) and yibirthday@126.com (Y.T.).

*These authors contributed equally to this work.

**S1 Supplementary method**

The proposed method has been described in the main text. Here we go into details on the process of BC update of Step 6 (in the main text), and rigorously analyze and demonstrate the validity of the whole proposed method. At the last, we give the rigorous analysis and derivation of computation complexity of the proposed method.

## S1.1 Process of BC update of Step 6

First, we describe the betweenness of a vertex and an edge. Let *G* = (*V*, *E*) be a (directed or undirected) graph, in which the number of vertices and edges are *n* and *m*, respectively. The distance *dG*(*s*, *t*) represents the shortest path that connects the vertex *s* and the vertex *t* in *G*. Then, the vertex betweenness for vetex *v*, i.e., *VB*(*v*), and the edge betweenness for the edge *e_u_*,*_v_*, i.e., *EB*(*e_u,v_*), can be respectively defined as *(1, 2)*

$VB(v)=\sum_{s,t\in v,s\neq t} \frac{\sigma_{st}(v)}{\sigma_{st}}$ (S1)

$VB(e_{u,v})=\sum_{s,t\in v,s\neq t} \frac{\sigma_{st}(e_{u,v})}{\sigma_{st}}$ (S2)

where *σ_st_* is the number of the shortest paths between vertices *s* and *t*, *σ_st_*(*v*) is the number of the shortest paths passing through *v* from *s* to *t*, and *σ_st_*(*e_u_*,*_v_*) is the number of the shortest paths passing through *e_u_*,*_v_* from *s* to *t*.

According to Corollary **S1**, the shortest path between any source *s* and any terminal vertex *t* of different communities includes two parts, i.e., the shortest path between the vertices in the HSN and the shortest path between the intercommunity vertices and other vertices of the same communities. Therefore, the length of the shortest path between any source *s* and any terminal vertex *t* of different communities can be calculated as follows:

$dG(s,t)=\{dG(s,g_{a,i})+dG(g_{a,i},g_{b,j})+dG(g_{b,j},t)\}_{\min}$ (S3)

where *C_a_* and *C_b_* are the communities containing *s* and *t*, respectively; $GV_{a}=C_{a}\cap HSN=\{g_{a,1},g_{a,2},...,g_{a,n_{a}}\}$ and $GV_{b}=C_{b}\cap HSN=\{g_{b,1},g_{b,2},...,g_{b,m_{b}}\}$ are the sets of the intercommunity vertices of *C_a_* and *C_b_*, respectively; *n_a_* and *m_b_* are the numbers of *GV_a_* and *GV_b_*, respectively, $s\in C_{a}$, $t\in C_{b}$, $g_{a,i}\in GV_{a}$, $g_{b,j}\in GV_{b}$, $i=1,2,...,n_{a}$, $j=1,2,...,m_{b}$.

Eq. **S3** indicates that any shortest path from *s* to *t* can be divided into three segments: from *s* to *g_a,i_* from *g_a,i_* to *g_b,j_*, and from *g_b,j_* to *t*. Therefore, an additional search for the shortest paths for all intercommunity vertex pairs in the HSN occurs in Step 3 (in the main text).

Then, the problem of the shortest path search from *s* to *t* can be transformed into the matching problem of a bipartite graph *GV* with the bipartition (*GV_a_*, *GV_b_*), and any matching *x_k_* should satisfy Eq. **S3**, as shown in Fig. **S1**. This problem is solved in Step 6 (in the main text). Supposing that the matching set *S* in *GV* consists of *X* elements, the *X* value is between 0 and *nm*, where *nm* is the number of the complete matches, and *X* is greater than 0 if the shortest paths from *s* to *t* exist. Supposing that $x_{k}=\{g_{a,i},g_{b,j}\}$ is any matching of the matching set *S*, the number of shortest paths passing through *x_k_* from *s* to *t*, *σ_st_*(*x_k_*) is given by

$\sigma_{st}(x_{k})=\sigma_{st}(g_{a,i},g_{b,j})=\sigma_{sg_{a,i}}\sigma_{g_{a,i}t}(g_{b,j})=\sigma_{sg_{a,i}}\sigma_{g_{a,i}g_{b,j}}\sigma_{g_{b,j}t}$ (S4)

Where $\sigma_{sg_{a,i}}$, $\sigma_{g_{a,i}g_{b,j}}$ and $\sigma_{g_{b,j}t}$ are calculated and stored in the communities *C_a_*, *C_b_* and HSN independently in Steps 5 and 3 (in the main text).

Therefore, the total number *σ_st_* of the shortest paths from *s* to *t* can be obtained as follows:

$\sigma_{st}=\sum_{k=1}^{X} \sigma_{st}(x_{k})$ (S5)

Accordingly, for those vertices and edges that lie on the shortest path from *s* to *t* that any matching *x_k_* lies on, their betweenness calculation for any matching *x_k_* can also be divided into three segments, including the first segment from *s* to *g_a,i_*, the second segment from *g_a,i_* to *g_b,j_*, and the third segment from *g_b,j_* to *t.* When the vertex *v* or the edge *e_u,v_* lies on the first segment, accumulative BC indexes Δ*VB*(*v*) and Δ*EB*(*e_u,v_*) for the vertices and edges, respectively, are given by

$\Delta VB(v)=\frac{\sigma_{sg_{a,i}}(v)\sigma_{g_{a,i}g_{b,j}}\sigma_{g_{b,j}t}}{\sigma_{st}}$ (S6)

$\Delta EB(e_{u,v})=\frac{\sigma_{sg_{a,i}}(e_{u,v})\sigma_{g_{a,i}g_{b,j}}\sigma_{g_{b,j}t}}{\sigma_{st}}$ (S7)

When the vertex *v* or the edge *e_u,v_* lies on the second or third segment, Δ*VB*(*v*) and Δ*EB*(*e_u,v_*) can be similarly determined by using Supplementary Eq. **S6** and Eq. **S7**, respectively.

Particularly, deduced from Supplementary Eq. **S5** and Eq. **S6**, the accumulative BC, i.e., Δ*VB*(*g_a,i_*) and ΔV*B*(*g_b,j_*) for the gate-vertices *g_a,i_* and, *g_b,j_* is given by

$\Delta VB(g_{a,i})=\Delta VB(g_{b,j})=\frac{\sigma_{sg_{a,i}}\sigma_{g_{a,i}g_{b,j}}\sigma_{g_{b,j}t}}{\sigma_{st}}$ (S8)

After the whole matching set *S* is traversed, for those vertices and edges that lie on the shortest path from *s* to *t*, their betweennesses have been accumulated by using the procedure. Step 6 (in the main text) is completed thoroughly until all vertex pairs of different communities are traversed.

## S1.2 Lemma and corollary

Here we give two lemmas and corollaries for the validation of our method.

**Lemma S1*:*** Given two intercommunity vertices *v_ga_* and *v_gb_* in the community *C_i_* of a network *G* with a hierarchical community structure, let *dC_i_*(*v*_ga_, *v*_gb_) be the shortest distance between *v_ga_* and *v_gb_*, which only lies within the community, and let *dHSN*(*v*_ga_, *v*_gb_) be the length of the shortest path between *v_ga_* and *v_gb_*, which only lies within the HSN. If $dC_{i}(v_{ga},v_{gb})<dHSN(v_{ga},v_{gb})$ is satisfied, then the shortest paths between vertices of the same community do not pass through the vertices of other communities. Otherwise, there must exist some vertex pairs of the same community whose shortest paths pass through other communities.

**Proof*:*** If a shortest path between two vertices (*s*, *t*) within a community *C_i_* does pass through the vertices of other communities, the shortest path must pass through two intercommunity vertices of *C_i_*. Let the two intercommunity vertices be *v_ga_* and *v_gb_*, then, [*dC_i_*(*s*,*v*_ga_,) + *dHSN*(*v*_ga_, *v*_gb_) + *dC_i_*(*v*_gb_, *t*)] < [*dC_i_*(*s*,*v*_ga_,) + *dC_i_*(*v*_ga_, *v*_gb_) + *dC_i_*(*v*_gb_, *t*)], namely, $dHSN(v_{ga},v_{gb})<dC_{i}(v_{ga},v_{gb})$. This contradicts the condition $dC_{i}(v_{ga},v_{gb})<dHSN(v_{ga},v_{gb})$. Otherwise, at least, there exist the gateway vertex pairs who satisfy the condition **■**

**Lemma S2*:*** For a network with a hierarchical community structure, the shortest paths between any two intercommunity vertices only pass through the vertices and edges of the HSN of the network.

**Proof*:*** For two intercommunity vertices in the same community *C_i_*, if the shortest paths between the two intercommunity vertices in a global network pass through other vertices and edges of the same community outside of the HSN, then the definition of HSN is contradicted. Otherwise, if the shortest paths between the two intercommunity vertices in a global network pass through other vertices and edges outside of the HSN of another community *C_j_*, then the shortest paths need to pass through community *C_j_* from two intercommunity vertices (or one gateway) of the community *C_j_*, and these vertices and edges in the shortest paths between the two intercommunity vertices of community *C_j_* must be in the HSN (HSN definition); then, the former condition contradicts the HSN definition. **■**

**Corollary S1*:*** The shortest path search from source *s* to terminal *t* through the HSN is equivalent to the shortest path search in the global network.

**Proof*:*** According to Lemma **S2**, the shortest path search between any two intercommunity vertices in the HSN of a network is equivalent to the shortest path search in the whole network. Then, *Corollary S1* is proved completely. **■**

**Corollary S2*:*** For any community *C_i_* that does not satisfy $dC_{i}(v_{ga},v_{gb})<dHSN(v_{ga},v_{gb})$ given two vertices (*s*, *t*) of *C_i_*, searching the shortest path between them within the global network is equivalent to searching the shortest path in the updated community *C*_3_′.

**Proof*:*** For the community *C_i_* that does not satisfy $dC_{i}(v_{ga},v_{gb})<dHSN(v_{ga},v_{gb})$, given the two vertices (*s*, *t*), if the shortest path between them passes through the vertices of other communities, the shortest path must go out from one of the two intercommunity vertices and comes back from the other according to the definition of intercommunity vertex. According to Lemma **S2**, the part of the shortest path outside the community must lie in the HSN. Since the part of the shortest path outside *C_i_* has been copied into the updated community *C*_3_′ (see the definition of updated community and the example in Fig. **S2**), according to Corollary **S1**, the shortest paths search between the two vertices within the global network is equivalent to the shortest path search in the updated community *C*_3_′. **■**

## S1.3 Mathematical analysis and proof

From the Lemmas and Corollaries, one can see that Lemma **S2** means that the shortest paths search for all intercommunity vertex pairs in the HSN is equal to the shortest path search in the global network. Therefore, the BC computation in the HSN is valid globally, as shown in Step 3 (in the main text).

Lemma **S1** indicates that for any community satisfying $dC_{i}(v_{ga},v_{gb})<dHSN(v_{ga},v_{gb})$, the shortest path searches in it are equivalent to those in the global network. Consistently, the relative BC computation in the community is equivalent to that in global network. Corollary **S2** shows that for any community not satisfying $dC_{i}(v_{ga},v_{gb})<dHSN(v_{ga},v_{gb})$, after updating the community, the shortest path can be searched in the updated community. Therefore, the BC can be calculated precisely within each community by using Steps 4 and 5 (in the main text).

According to Corollary **S1** and Eq. **S3**, the shortest path searches between any source *s* and any terminal vertex *t* of different communities is valid globally, thus the relative BC computations are correct consistently, as described in Step 6 ( in the main text) and BC update of Step 6 (in Section **S1.1**).

From the above mathematical analysis and proof, it is shown that the proposed method described by Steps 1-6 (in the main text) is rigorous and valid, and can be applied to any networks with hierarchical community structure, inspective of any community detection methods.

**S2 Supplementary Figure**


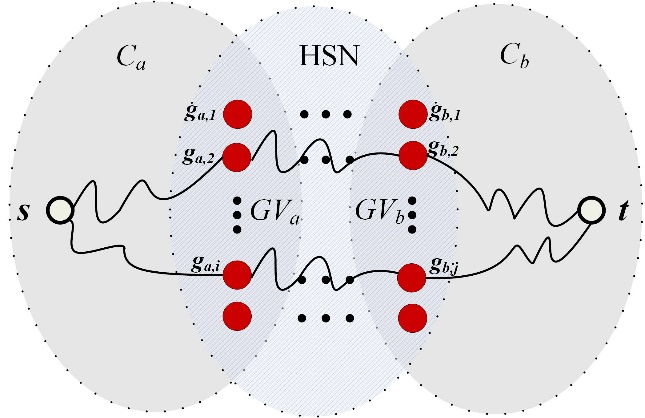


**Figure S1. The matching set *S* consists of all vertex pairs *x*_k_ = {*g*_a,i_, *g*_b,j_} that satisfy Eq. S3.** The nodes *s* and *t* are the source vertex and terminal vertex, respectively, and *C_a_* and *C_b_* are the communities containing *s* and *t*, respectively. The shortest path of the vertex pair is strongly related to the intercommunity vertices of communities *C_a_* and *C_b_*, as shown in Eq. **S3**.


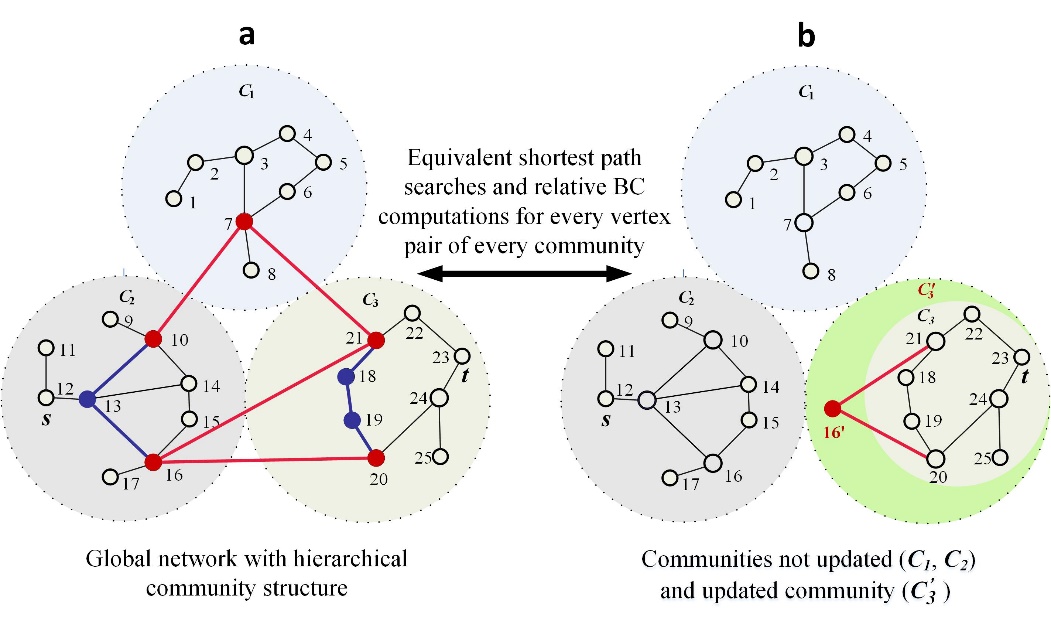


**Figure S2. Equivalent shortest path searches and relative BC computations for every vertex pair within each community**. (a) The global network with hierarchical community structure. (**b**) The updated community (*C_3_*′) and the communities (*C_1_*, *C_2_*) that need not been updated.

**
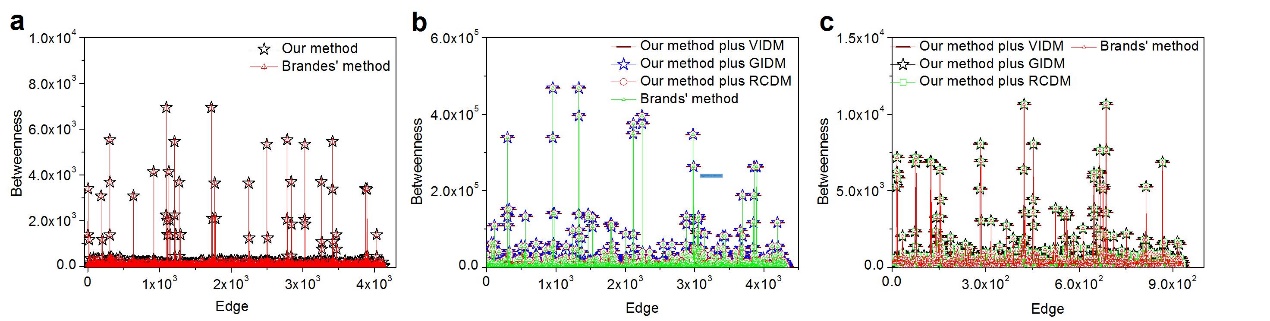
**

**Figure S3.** **Calculation validity of edge betweeness**. (**a**) The betweenness of each edge in the artificial network. (**b**) The betweenness of each edge in the Gansu power grid. (**c**) The betweenness of each edge in the Henan power grid.

**S3 Supplementary References**

1. Kourtellis, N., Morales, G. D. F. & Bonchi, F. Scalable online betweenness centrality in evolving graphs. *IEEE Trans. Knowl. Data. En*. 27, 2494-2506 (2015).
2. Puzis, R., Zilberman, P., Dolev, S., Brandes, U. Topology manipulations for speeding betweenness centrality computation. *J. Compl. Netw*, 3, 84-112 (2015).
3. Tyler, J., Wilkinson, D. & Huberman, B. Automated discovery of community structure within organizations. Information Society 21, 143-153 (2005).
